# Fostering Experiential Learning and Evidence-Informed Impact in Health Systems: Reflections From a Canadian Health System Impact Fellow

**DOI:** 10.34172/ijhpm.8074

**Published:** 2024-08-28

**Authors:** Jawad Chishtie, Peter Kellett, Mohammed Imran, Meaghan Sim, Shawna Cronin, Christopher Collins, Beverley Catharine Craven, Sara Guilcher, Susan Jaglal

**Affiliations:** ^1^Rehabilitation Sciences Institute, University of Toronto, Toronto, ON, Canada.; ^2^Alberta Health Services, Edmonton, AB, Canada.; ^3^Faculty of Health Sciences, University of Lethbridge, Lethbridge, AB, Canada.; ^4^Allama Iqbal Open University, Islamabad, Pakistan.; ^5^Nova Scotia Health Innovation Hub, Halifax, NS, Canada.; ^6^University of Toronto, Toronto ON, Canada.; ^7^Faculty of Science, Ontario Tech University, Oshawa, ON, Canada.; ^8^KITE, Toronto Rehabilitation Institute-University Health Network, Toronto, ON, Canada.; ^9^Division of Physical Medicine and Rehabilitation, Department of Medicine, University of Toronto, Toronto, ON, Canada.; ^10^Rehabilitation Sciences Institute, Faculty of Medicine, University of Toronto, Toronto, ON, Canada.; ^11^Leslie Dan Faculty of Pharmacy, University of Toronto, Toronto, ON, Canada.; ^12^Institute of Health Policy, Management and Evaluation, University of Toronto, Toronto, ON, Canada.; ^13^Department of Physical Therapy, University of Toronto, Toronto, ON, Canada.

## Background

 Classically, the concept of an embedded scholar is of a team member who maintains an academic affiliation in a health system organization, which is lately being viewed as a pragmatic shift towards creating evidence-informed impact.^1,2^ Towards this goal, the Health System Impact (HSI) Fellowship is one of the Canadian Institutes for Health Research’s (CIHR’s) flagship programs, that cultivates academic and health system organization partnerships, while catalyzing development of learning health systems (LHS). The program supports developing emerging leaders’ professional competencies and potential for evidence-based impact outside the traditional university environment.^3^

 In this article, I reflect on my experience as an embedded doctoral fellow, considering how to foster a mutually beneficial pathway for addressing real-world problems that demand pragmatic problem-solving skills. The views expressed here are informed by judicious feedback by expert researchers in health systems, adding to the body of literature on embedded scholarship arrangements.^4,5^

 I worked as a HSI Fellow at the Canadian Institute for Health Information (CIHI), as part of their Analytic Techniques and Tools team, Advanced Analytics Branch in partnership with the University of Toronto from September 2019 to December 2020. My main project was to develop an interactive visualization (IV) dashboard for the Population Grouping Methodology (Pop Grouper), making the digital health product more accessible to non-technical users. The dashboard was viewed as a digital health tool for operationalizing a rapid LHS, promoting the use of population health data for assessing health status and profiles for planning and delivering health services. The article does not provide the details of the dashboard, rather focuses on reflections of my embedded fellowship experience on co-designing a visualization-based application.

###  Settings and Use Case

 CIHI is a federal non-profit that aims at accelerating evidence-based improvements in healthcare, health system performance and population health.^6^ The Pop Grouper offers a case-mix classification using person-level demographic and clinical data, categorizing the national population into 226 major health conditions and profiles.^7^ Based on these profiles, the Grouper supports health service planning and delivery.

 One of the known limitations with the existing Pop Grouper is the coding expertise required for analyzing data, which poses a hurdle for non-technical users. The major advantage of IV products is that they promote users’ analytic reasoning, while reducing cognitive load of complex data.^8^ To facilitate access for non-technical users, the Case Mix team was interested in developing an IV dashboard for the Pop Grouper as a complement to the current offering, while testing the SAS Visual Analytics suite for developing the product, and further use.^9^ The use-case dataset comprised two-year health data of over 10 million cases from a Canadian jurisdiction.

###  Conceptual Frameworks and Work Phases

 From March to October 2019, I engaged in iterative rapid prototyping for co-designing a low-fidelity dashboard, using Börner’s Data Literacy Framework,^10^ while employing Spinuzzi’s^11^ approach dividing co-design phases into exploration, discovery and prototyping. The process was underpinned by the pragmatic world-view, which posits that truth is what works at the time, thereby allowing flexibility towards addressing objectives, engaging in knowledge generation in its context.^12^ Knowledge was assumed to be tied to practice, while the dashboard was created keeping in mind the value of it for the future.^13^ *Prototyping* is a powerful design method, cited as “worth a thousand meetings,”^14^ that served a key role in formalizing a vague idea and facilitating consensus.^15,16^ Further, data collection and analysis related to the co-design process was informed by a qualitative approach.^17,18^ Co-design participants included four CIHI staff, with oversight from the academic doctoral supervisory committee. Being an internal project using de-identified data, there were no ethical concerns.

**Figure 1:**
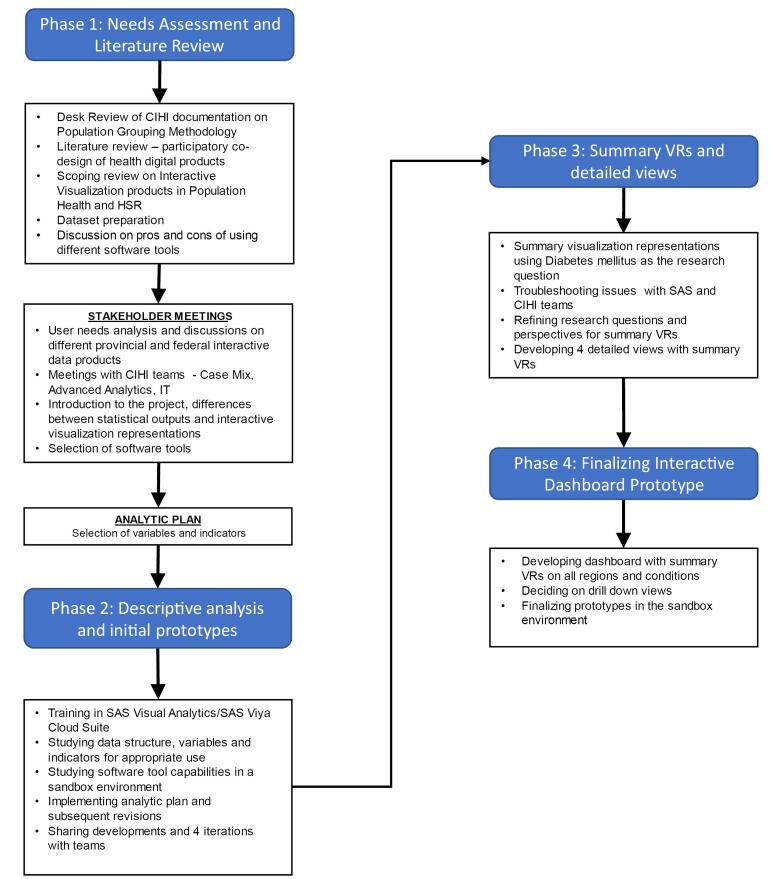
Phases of Work, Duration and Main Tasks During the Visualization Representation Prototype Design Process. Abbreviations: VRs, visualization representations; IT, information technology; HSR, Health Services Research; CIHI, Canadian Institute for Health Information.

**Figure 2:**
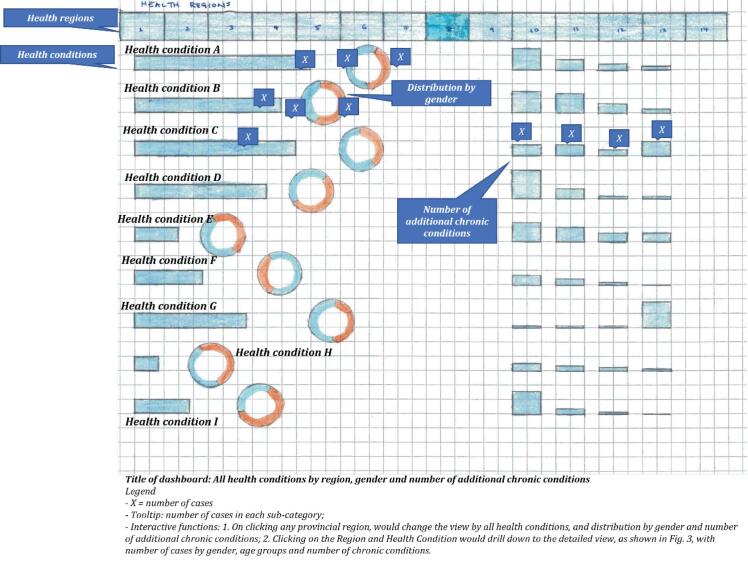
Prototypical Interactive Visualization Dashboard (figure does not show actual data analysis).

 During the *exploration *phase, I reviewed literature on similar digital health products, held extensive consultation sessions to elicit and agree on team needs, and developed analytic plans. During the *discovery *phase, plans were fine-tuned, while visualizations were developed to promote understanding of the dataset and functions of the software. During the *prototyping *phase, three iterations of the dashboards were developed and tweaked with two final views: an overall summary presenting 226 conditions; and drill down capability to show population level metrics for each condition. The process is illustrated in Figure 1. Feedback from CIHI teams were gathered on a continual basis and analyzed using thematic analysis, arriving at five key considerations related to: refining the analysis; building interactivity functions; and integrating views. The final view of the dashboard is presented as a hand drawing, without actual data (Figure 2). The dashboard presented 226 health conditions across more than 10 jurisdictions. The individual elements in the visualizations allowed further drill down ability for each health condition. The second drill down view presented health demographics by sex, chronic conditions, and age groups. In essence, the dashboard greatly simplified complex data into two views.

## Reflections on Impact and Future Directions

###  Value-Add From the Opportunity to Co-design the Application 

 Rapid iterative prototyping is an accepted method for IV products in healthcare.^19,20^ I used a flexible methodology which can serve as a potential model for the development, implementation, and honing of visualization-based analytic solutions within large publicly funded healthcare organizations.

 For the embedded fellow, development of the digital product was a rare, unparalleled opportunity as an HSI Fellow for application co-design with varied CIHI teams including information technology, Case Mix and the Advanced Analytics teams. The project was collaboratively envisioned at every stage, with thorough discussions involving the CIHI teams. This was done to shape an innovative project with significant impact, in line with the organization’s mandate.

 To the best of our knowledge, this was the first IV application developed using Pop Grouper data reported in peer reviewed literature.^8,21^ The dashboard greatly simplified complex health data, bringing together more than 3000 data elements in one comprehensive view, while the second drill down view presented more than 40 data elements related to population demographics. The prototype was viewed as an opportunity for making complex data accessible to non-technical users. Interacting with such large number of metrics in a user-friendly visual format allows users to explore data, conduct analysis in a non-linear fashion, and gain insights.^22^ Such data driven initiatives allow operationalization of rapid LHS, gathering insights for iterative improvement in services and care delivery.^23,24^

###  Role of an Embedded Fellow: Expectations and Limits

 Given the appeal of an *ivory-tower-meets-trenches* arrangement, embedded scholarship has been experimented in various sectors.^2^ In health systems, Sim et al^5^ suggest the role a fellow can be as a “central agent” and “a conduit for system level change.” However, given that organizational culture being a known, major challenge,^2^ suggesting such system-wide change can be a considerable undertaking. My experience suggests that expectations need to be realistic for a Fellow and that *collaboration* and *facilitation* are better suited objectives as has been pointed out by other experts.^25^ This is particularly true for the one-year doctoral fellowship, which is a limited time span to initiate even the smallest system level change.

 I was inclined towards creating long-term impact by developing a product that could inform future work. My personal growth while working with teams was acting as a *data strategist* to guide the appropriate use of data, co-design applications, and advise future digital health products.

###  Navigating Competing Priorities and the Co-design Process

 Embedded fellows occupy an often-elusive *middle ground* within and between organizations, with multiple competing requirements.^2,26^ For me, this translated in managing expectations between organizations, supervisors and the thesis committee. This proved to be the most challenging facet of the Fellowship. In addition, decision-making processes within CIHI were entirely different from a university. Such embedded arrangements could be a sharp departure from independent academic work. Further, applied co-design can prove demanding in interdisciplinary settings.^20^ Being an embedded fellow helped convey project requirements and changes in scope between CIHI teams to allow adaptability in achieving project objectives.

 Iterative design can have its challenges, such as stakeholder disagreement on the level of detail.^20^ Such issues are expected when stakeholders have different backgrounds and requirements.^20^ As part of the CIHI teams, I had the opportunity to reach an understanding on the expected outputs and limitations well in time. Being embedded in the team also helped reduce lengthy deliberations, allowing efficient troubleshooting.

 One of the major learnings from the Fellowship was realizing the value of building close, interdependent collaborations with multiple teams and individuals in organizational settings. This required proactive, open communication with supervisors, and teams, to collaboratively problem solve as issues arose. One particular insight was the need to invest time in the initial phases to develop a detailed study protocol as a living document for the thesis committee members and CIHI teams for a continued, shared understanding throughout the course of the Fellowship.

 The dual mentorship of both academic and health system supervisors has proven impact for the HSI Fellowship.^27^ In this regard, I found that the role of the academic supervisor was most crucial, especially for doctoral candidates where thesis requirements is a prime concern. My academic supervisor took on an active role, holding bi-weekly joint meetings to assess progress, discuss issues for fulfilling academic and host organization requirements, and provide guidance for keeping the project on track.

###  Fellowship Outputs

 Scholarship, by design is to be shared for being critiqued, through peer-reviewed papers, reports and presentations. This expectation is inherent for doctoral and post-doctoral fellows alike. Ideally, an embedded scholar co-produces jointly owned, “*decision-relevant, impact-based” *products.^2,28^ During my Fellowship, outputs included a scoping review of IV applications, analytic plans and prototypical dashboards. However, I could not publish the results from the dashboard due to organizational policies. For doctoral fellows, this could be a concern for fulfilling thesis requirements. Hence, scholarly outputs can be agreed on early in the Fellowship, so that necessary approvals are in place for publishing results.

###  Organizational and Candidate’s Readiness 

*Readiness* is used as an axiom for consideration to the opportunity and the challenges that embedded fellowship arrangements entail, for both the candidate and the host organization. Organizations typically seek skill, in-depth knowledge, and rapport building from scholars, while personal background and career goals are important considerations for fellows in meeting the expected role.^29^ These expectations are to be taken seriously, as the Fellows’ engagement is limited.

 While health organizations genuinely desire evidence-informed change, there is a need for creating opportunities, increase organizational tolerance to disruption, and work towards addressing the divide between scholarship and operations.^26,30^ This can be viewed as a shared responsibility of senior organizational leadership. In my experience, disruption can be well tolerated, but difficult to negotiate simply due to existing business rules.

###  Fostering a Mutually Rewarding Experience

*How can a mutually rewarding experience be fostered for both the health system organization and the HSI Fellow? *In an attempt toanswer this complex question, I distill lessons from the literature and my experience into a checklist for host organizations and potential candidates to critically assess their position and alignment to the Fellowship’s goals. The five categories of the checklist include Personal Characteristics, Academic and Professional Development, Resources, Long-term goals, and Contingency Planning (Table). Some specific points are elaborated here.

**Table:** Checklist for Potential Candidates and Host Organizations to Consider for an Embedded Scholarship Arrangement

**Aspect**	**Reflective Questions for the Candidate**	**Reflective Questions for the Host Organization**
Personal characteristics	Does this opportunity align with my personal values? Does this opportunity align with my personal goals? Will my work style align with my host system and academic supervisors? What are the best ways to communicate and manage expectations between different institutions, supervisors and other involved individuals? Is there written agreement on the frequency and mode of communication?	Does the fellow's personal characteristics fit with the overall team culture? Does the host system supervisor's work style align with the candidate's and the academic supervisor?
Academic and professional development	(For doctoral candidates) To what extent does the opportunity align with my doctoral work? If there is no alignment, what other aspects will I gain from? Is there an existing project that I will work on? Am I to write up a new project? Is there time to write up a proposal for agreement with the host and academic institutions? Is a brief proposal written and agreed with the supervisors? Does the host organization want me to help out with other projects? Is there a clear plan for professional development? Will professional development opportunities interfere with my academic work? Can I afford to do that? Can I commit to the time needed to coordinate the project and manage expectations between the host and academic supervisors, and if applicable, the academic committees?	Do we want the candidate to contribute to existing projects? Do we want the candidate to develop a new project? (For doctoral candidates) Does the host organization allow data access and publication of results? Can these be included in the thesis? Are we viewing the candidate as a potential team member, or as an intern to gain experience? How and to what extent does the candidate's project align with the larger goals of the organization? Are there clear objectives, methods and outcomes clearly defined for the project? Is there a clear understanding for the candidate's professional development opportunities within and beyond the host organization? What is the time commitment expected from all parties? Does the host system supervisor and the academic supervisor, and the thesis committee, if applicable, have time to supervise this new project?
Resources	Am I interested in the Fellowship to fund my education and living expenses? (For doctoral candidates) Will the expenses work out for me? Have I considered that my existing funding may be deferred or cancelled, such as supervisor and graduate unit funds? Have I weighed the lost earnings against the opportunities from the fellowship? Will I have to forgo other financial opportunities due to lack of time? (For doctoral candidates) Do I have the resources to fund my education, if this arrangement does not work out?	Do we have funding available for the embedded arrangement, and other unforeseen expenses for the candidate's project? Are we interested in bringing on a candidate to leverage the funding opportunity? Do we have resources lined up for the potential project?
Long term goals	Does this opportunity align with my long-term career goals? Is there an opportunity to work with the organization beyond the Fellowship?	Is the candidate a potential employee for the team? What is the value-add from the candidate's skills and project?
Contingencies	Do I have an alternate project, in case of problems? Does the host and academic institutions agree to the contingency plan or project?	Is there an alternate project identified? Are resources lined up for the alternate project? Is there agreement with the candidate on the contingency plan or project?

 The onus for undertaking an embedded experience lies with the candidate. The role of the academic and host system supervisors is key, being the main links to both institutions, the combined policies of which would guide the work of the candidate. For doctoral candidates, it is of utmost importance to assess the alignment with their doctoral work, and views of the academic committees, the graduate unit and their supervisors. This assumes greater importance if they are funded by their supervisors or the graduate unit. Further, project and academic deliverables should be outlined and agreed at the outset, and progress jointly tracked according to a plan, for academic and non-academic outputs. Further, it is imperative that projects are aligned with both the interests of the fellow and the host organization, while contingency planning is needed in case the project or any of its parts do not fall into place. It is important to reflect on the extent that personal, academic and career goals align to the opportunity presented by the host organization. This could mean communicating early and openly about these goals with host system and academic supervisors.

 For the host supervisor and organization, it is important to appreciate the middle ground that the embedded fellow partakes within and between organizations. While host organizations may genuinely desire incremental cultural change, it can be accompanied with disruption. However, such disruption can create opportunities for evidence-informed change, if the recipe is done justice with the ingredients of appropriate resources, support and proactive planning. Time should be allocated for reaching a detailed understanding on the scope of work, gathering resources, and training on internal systems for the fellow to appreciate complex decision-making. Data access and publication of results should be worked out before, during and after project closure and when the candidate leaves the organization. In addition, it would be worthwhile to ensure that organization teams are ready to utilize the scholar’s skills, which may require approvals from higher management. All these aspects point to a need for host organizations to prepare in advance to help realize the potential of embedded fellowships.

## Limitations

 While this paper attempts at bridging a gap in literature on pragmatic experiential learning experiences, there are important limitations. Firstly, stakeholder feedback for verification or triangulation of views is not presented due to organizational policies. However, throughout the article, a balanced view of the challenges and opportunities, tempered with judicious feedback from supervisory committee experts on the co-design process, experiential learning, and aspects on aligning interests of the scholar, academic and host organization is presented. The organization periodically reviewed and validated the process and results of the prototyping exercise. Although the dashboards were co-designed, views shared do not reflect those of the host or academic organization.

## Conclusion

 This experience showed that co-creation with an embedded scholar can prove valuable in promoting innovative digital health products for healthcare agencies to inform service planning. An enabling environment, realistic expectations on the part of the academic and host organizations, and reflexivity from the scholar form the essential ingredients for fostering a mutually rewarding fellowship experience. A checklist with reflective questions for the fellow and host institution was developed to support these arrangements. Future research is needed to explore the opportunities and challenges faced by health system organizations and scholars working in embedded arrangements.

## Acknowledgements

 We thank the CIHI teams for participating in the co-design of the IV dashboard, and experts from various disciplines on advising the embedded fellowship project.

## Ethical issues

 Ethical review was not required for this research. No data on human subjects is being presented. An ethical concerns section details these aspects in the manuscript.

## Conflict of interests

 Authors declare that they have no competing interests.

## Disclaimer

 Acknowledgement of CIHI and/or CIHI staff in this publication does not imply CIHI’s endorsement of its interpretation of data, opinions or conclusions.
